# Hydrocarbon Transportation in Heterogeneous Shale Pores by Molecular Dynamic Simulation

**DOI:** 10.3390/molecules29081763

**Published:** 2024-04-12

**Authors:** Shuo Sun, Mingyu Gao, Shuang Liang, Yikun Liu

**Affiliations:** 1 Department of Petroleum Engineering, Northeast Petroleum University, Daqing 163318, China; 2 Key Laboratory of Enhanced Oil Recovery, Northeast Petroleum University, Ministry of Education, Daqing 163318, China; 3 The No.2 Oil Production Plant of Northwest Petroleum Bureau, Kuche 842000, China

**Keywords:** heterogeneous shale, molecular dynamics, oil–water two-phase region, fluid behavior, interaction energy

## Abstract

Shale oil in China is widely distributed and has enormous resource potential. The pores of shale are at the nanoscale, and traditional research methods encounter difficulty in accurately describing the fluid flow mechanism, which has become a bottleneck restricting the industrial development of shale oil in China. To clarify the distribution and migration laws of fluid microstructure in shale nanopores, we constructed a heterogeneous inorganic composite shale model and explored the fluid behavior in different regions of heterogeneous surfaces. The results revealed the adsorption capacity for alkanes in the quartz region was stronger than that in the illite region. When the aperture was small, solid–liquid interactions dominated; as the aperture increased, the bulk fluid achieved a more uniform and higher flow rate. Under conditions of small aperture/low temperature/low pressure gradient, the quartz region maintained a negative slip boundary. Illite was more hydrophilic than quartz; when the water content was low, water molecules formed a “liquid film” on the illite surface, and the oil flux percentages in the illite and quartz regions were 87% and 99%, respectively. At 50% water content, the adsorbed water in the illite region reached saturation, the quartz region remained unsaturated, and the difference in the oil flux percentage of the two regions decreased. At 70% water content, the adsorbed water in the two regions reached a fully saturated state, and a layered structure of “water–two-phase region–water” was formed in the heterogeneous nanopore. This study is of great significance for understanding the occurrence characteristics and flow mechanism of shale oil within inorganic nanopores.

## 1. Introduction

Global energy transition trends have emerged in various countries [1], but there is still significant uncertainty and multi-selectivity in the path and pace of transformation. According to the prediction of the International Energy Agency (IEA) and OPEC in 2020, the world’s oil demand will not be less than 100 million barrels per day by 2040 and will remain stable for a long period of time. Therefore, it remains challenging to fully replace the demand for oil. Moreover, oil and natural gas will still play an important role in transforming from fossil fuels to clean energy systems. As an important alternative energy, shale oil possesses enormous resources. The resource evaluation results of 157 shale oil enrichment formations in 116 basins worldwide reveal that the technically recoverable resources of shale oil are about 251.2 billion tons. Among them, the technically recoverable resources of low-maturity and medium- to high-maturity shale oil are 209.9 billion tons and 41.3 billion tons, respectively. The United States is the first country to realize the commercial development of shale oil, and its marine shale oil features excellent continuity, relatively greater thickness, and a higher reservoir pressure coefficient. The shale oil resources of Argentina rank fourth in the world, which belong to marine shale. The thickness of typical shale oil reservoirs in Argentina is greater than that of most oil and gas development areas in the United States. China’s shale oil is a typical lacustrine deposit with enormous potential in medium- and low-maturity resources [2,3]. It is estimated that the economically recoverable resource is 20–25 billion tons under the Brent oil price of USD 60–65 per barrel. Shale is characterized by low brittleness, challenging fracturing, a well-developed fracture network [4], strong heterogeneity, and a high clay mineral content [5]. Scanning electron microscopy (SEM) has confirmed the presence of organic pores, inorganic pores, and fractures in shale reservoirs, and the kerogen organic pores range from 2 to 100 nm. The pore size distribution results of the Wufeng–Longmaxi Formation shale in the Fuling area, Sichuan Basin, showed that the organic pores mainly distribute between 2 and 50 nm, and inorganic pores range from 4.03 to 500 nm, with the majority of inorganic pores ranging from 2 to 50 nm. Curtis et al. [6] used high-resolution EMG to scan the shale clay pores, and the results showed that the pores within the clay exhibit a “slit-like” shape, with a width mainly ranging from 3 to 20 nm. Loucks et al. [7] observed a variety of nanopores with pore sizes ranging from 5 to 750 nm in the Barnett shale, and the statistical results showed that pores at 5–15 nm dominate the frequency of pore size distribution.

It is difficult to describe the fluid behavior in such small pores, especially inorganic and clay pores. Currently, the study methods for nanoscale fluid behavior can be divided into experiments, theoretical models, and molecular simulations. A laboratory experiment is a reliable technology for studying fluid behavior; microfluidic and nanofluidic devices are used as visualization models of complex porous media. Wang et al. [8,9,10] studied alkanes, alkane mixtures, multi-phase flow, and phase behavior in microchannels and nanochannels. Zhang et al. [11] fabricated a dual-scale microfluidic/nanofluidic channel with a depth of 250 nm to study the multi-phase flow in “shale-like” unconventional dual-porosity tight porous media. Low-field nuclear magnetic resonance (NMR) is a rapid non-destructive detection technique for investigating hydrogen-bearing fluid phases (e.g., water and methane) in porous media [12]. It is widely used in the reservoir characterization and fluid (oil and water) saturation measurement. Liu et al. [13] used NMR for the first time to investigate the changes in adsorbed and non-adsorbed methane during the injection of CO_2_, which is of great significance for evaluating the potential of CO_2_ injection to enhance the recovery of adsorbed gas in a shale gas reservoir. Zhu et al. [14] carried out NMR-based CO_2_ miscible displacement experiments on oil-saturated shale and sandstone; the recovery of shale oil under different occurrence states was obtained. In addition, Javadpour et al. [15] measured the slip length of liquid flow on the shale surface using atomic force microscopy (AFM). Most of those experiments use artificial porous materials (such as glass, silicone, polydimethylsiloxane, etc.) and mainly rely on hypothetical models. For example, microfluidic chips have only one dimension of nanopores, which differ tremendously from the real shale surfaces in physical and chemical properties. Moreover, experimental samples are commonly prepared and measured at room temperature (25 °C, 30 °C) [16,17], which causes difficulty in reflecting the actual formation conditions. Furthermore, it is difficult to observe fluid transport and adsorption within shale micropores (less than 2 nm) effectively using current laboratory techniques [18,19].

Several theoretical models have been developed to describe the fluid behavior in shale nanopores. It is common to describe the transport of shale oil in nanopores with a flow enhancement model considering the slip length [20]. The flow enhancement formula integrates the influence of solid–liquid interaction on adhesion and surface diffusion. Mattia et al. [21] derived a model for the slip velocity of water flow in carbon nanotubes (CNTs) and thus obtained a flow enhancement model that considers the surface roughness and mixed wettability on the flow, but the fitting accuracy of the model is relatively low and does not consider adsorption. Moreover, the application of Mattia’s model in studying the oil flow in CNTs has not been validated until now. Cui et al. [22] proposed a flow enhancement model that incorporated boundary slip and adsorption of organic matter nanopores, in which the adsorbed oil and bulk oil were considered, respectively. The interface properties among organic and inorganic nanopores are different, resulting in different wall–fluid interactions. Zhang et al. [23] proposed an apparent permeability model based on flow enhancement to describe the oil transport in organic and inorganic nanopores. The results showed that the oil velocity in inorganic pores is much higher than that in organic nanopores. Based on the relationship of the slip length and contact angle. Wu et al. [24] obtained a generalized formula for the water phase slip length–contact angle and calculated the contact angle range from 0°to 150° and the slip length range from 0.1 to 22.8 nm. However, theoretical models sometimes have overly ideal simplification conditions, resulting in significant deviations from actual shale samples; the parameters in the model are difficult to obtain, and the accuracy of the model has not been fully verified.

**Figure 1:**
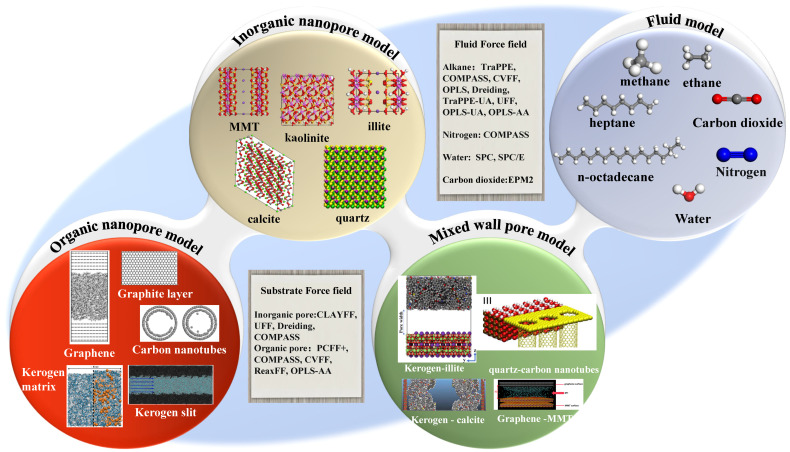
Summary schematic diagram of molecular dynamics studies on fluid behavior in different nanopores using various force fields.

Molecular dynamics simulation (MDS) is based on classical Newtonian mechanics, which calculates the motion coordinates of each particle in the system at each time step, optimizes the total energy of the molecule, and obtains the stable configuration and thermodynamic properties of the system [25]. Holt et al. [26] reported the flows of gas and water in CNT with a diameter less than 2 nm and found that the measured water flow rate was comparable to the flow rates inferred from MDS. In recent years, MDS has played a significant role in studying fluid behavior in shale pores. Sun et al. [27], Nan et al. [28], and Zhang et al. [29] listed the molecular dynamics studies of fluid behavior in different pores using various force fields. Figure 1 clearly presents the common physical models of shale and fluids. The organic matter in shale is kerogen, which is divided into three types according to the atomic (H/C) vs. (O/C) diagram, and kerogen in different shale plays may have different elemental ratios and functional groups. Ungerer et al. [30] created representative kerogen models using MDS based on the experimental results reported by Kelemen et al. [31]. Tesson et al. [32], Lukas et al. [33], Wu et al. [34], and Pan et al. [35] analyzed the adsorption and diffusion of CH_4_ and CO_2_ in kerogen, as well as the expansion of kerogen based on these models. Kerogen is usually regarded as an inelastic and rigid matrix, but it is essentially flexible. Firoozabadi et al. [32,36] confirmed that the flexibility of kerogen has a significant impact on gas adsorption and kerogen expansion; they found that gas adsorption in the flexible kerogen matrix is higher than that in the rigid kerogen matrix. Various force fields are used to describe kerogen, including classical CVFF, PCFF+, and COMPASS [37,38,39], as well as ReaxFF [40], that consider chemical reactions. Due to the complexity of the real molecular models of the shale organic matter, graphene and CNT are often selected as initial models for kerogens; the force field CVFF [41] and OPLS-AA [42] are usually used to describe the adsorption of alkanes on graphene surfaces.

The main inorganic matter of shale includes quartz, calcite, and clay minerals, and the clay minerals mainly include illite, montmorillonite (MMT), kaolinite, etc. [43,44]. Zheng et al. [45] studied the oil transportation under the influence of surface roughness and electrostatic interaction in quartz nanopores. Spera and Franco [46] focused on the diffusion for methane–ethane mixtures within calcite nanopores and found that the presence of ethane changed the self-diffusion of methane. In most plays, clay nanopores account for more than 50% of the volume of shale matrix [47]. In the Songliao Basin of China, the clay mineral content is 35–55%, and in the Sichuan Basin, the average clay mineral content is 42.1%. Clay minerals are prone to hydration expansion when encountering water, which may be the fundamental reason why hydraulic fracturing is not always effective. Clay minerals in shale are mainly illite and MMT [48,49,50]. Xiong et al. [51] and Liu et al. [52] investigated the formation of water bridges in illite nanopores and its impact on fluid transport. Chen et al. [53,54] studied the adsorption mechanism of CH_4_ and CO_2_ in K-illite slit pores. Wang et al. [55,56] and Yang et al. [57] studied the adsorption behavior of pure CO_2_, CH_4_, and CO_2_/CH_4_ mixture gases and the shale oil diffusion in MMT nanopores. The commonly used force fields for inorganic materials are CLAYFF, UFF, COMPASS, etc.

It can be concluded that most of the research on shale focuses on its organic or inorganic matrix, with a primary emphasis on the shale gas. The main investigations revolve around the adsorption and diffusion of shale gas, as well as the influence of temperature, pressure, and other factors. There is comparatively less research on shale oil. However, shale formation is a heterogeneous system consisting of complex composition and is essentially composed of several mixed wetting pores [58,59]. Given the prevalence of mixed wetting pore systems in shales, Hantal et al. [60,61] utilized the ReaxFF force field to study the influence of the interface bond formation reaction of organic porous carbon–inorganic matter (quartz and clay) on the interface, as well as the mechanism of interface failure, but did not describe the solid–fluid interaction. Lee et al. [62] constructed a composite membrane containing hydrophilic quartz and hydrophobic CNT and studied the activated desorption of hydrocarbon; the results indicated that the interfacial effects on wet kerogen surface hindered the recovery. According to the energy barrier results, CO_2_ or propane can replace water and effectively displace methane within the pores as there is no energy barrier inhibiting extraction in this case. Li et al. [63] built three composite shale models of kaolinite–kerogen IID, MMT–kerogen IID, and calcite–kerogen IID and explored the adsorption among CH_4_ and injection gases (CO_2_/N_2_) in various composite shale materials. They found that organic matter is the decisive factor of shale adsorption. Chen et al. [64] proposed a graphene–MMT pore as a shale matrix and investigated the adsorption characteristics of CH_4_/CO_2_. They confirmed that the graphene surface exhibits a significantly stronger adsorption capacity than MMT, especially for the adsorption of CO_2_. In addition, Yang et al. [65] and Dawass et al. [66] constructed kerogen–kaolinite composite pores to predict the adsorption of shale oil and gas, respectively.

**Figure 2:**
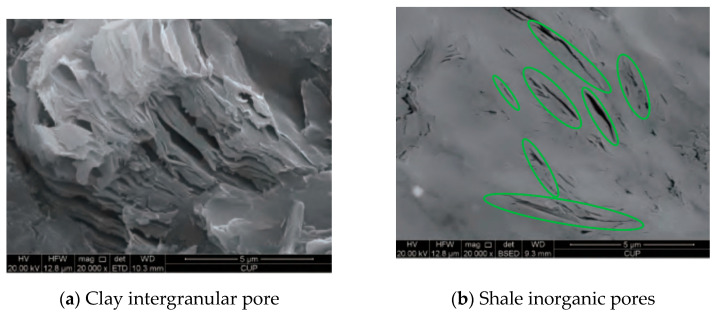
SEM images of inorganic and clay pores [67].

Therefore, the current research on mixed wetting and heterogeneity mainly focused on organic–inorganic composite walls. As the main component of shale, inorganic matters possess a relatively high specific surface, Figure 2a shows the intergranular pores of clay, which can be seen to have a relatively high specific surface area. Figure 2b shows the inorganic pores of shale, with green marked inorganic pores in the shape of narrow slits, which may affect fluid transport in shale reservoirs. Various inorganic materials exhibit notable differences in structure and thermodynamic properties, resulting in surface heterogeneity impacting the fluid flow in nanopores significantly. Therefore, based on a previous study by the research group (XRD results), we combined quartz and illite into inorganic composite shale, explored the fluid transportation in different regions of heterogeneous shale pores, and revealed the influence of various factors on fluid flow in heterogeneous shale pores.

## 2. Results and Discussion

### 2.1. Fluid Distribution and Transportation in Heterogeneous Shale Pores

**Figure 3:**
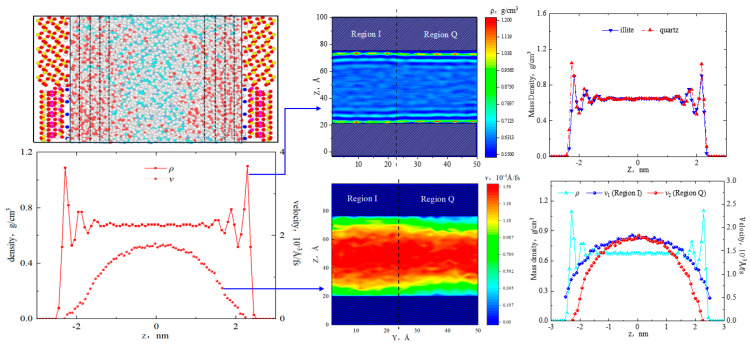
Octane microstructure and density and velocity distribution in 5 nm pores.

Figure 3 shows the last snapshot of n-octane after the equilibrium in the heterogeneous pore (width of 5 nm at 360 K, 30 MPa, and 0.002 Kcal/(mol∙Å)) with the mass density and velocity distribution in the Z direction. When calculating the mass density, the unit length of the Z direction was 0.08 nm, which was significantly smaller than the diameter of the n-C_8_ H_18_ molecule (0.63 nm). Octane molecules exhibited non-uniform distribution in the heterogeneous inorganic pore. Due to the interaction with the pore surfaces, the density fluctuation of octane molecules was higher in the near-wall region, decreased in the far-wall region, and remained almost unchanged at the center of the pore. The average density was 0.680 g/cm^3^, calculated by taking the arithmetic average of the density between z = −0.50 nm and z = 0.50 nm, which was basically consistent with the experimental results (0.67996 g/cm^3^) published by the National Institute of Standards and Technology (NIST).

**Figure 4:**
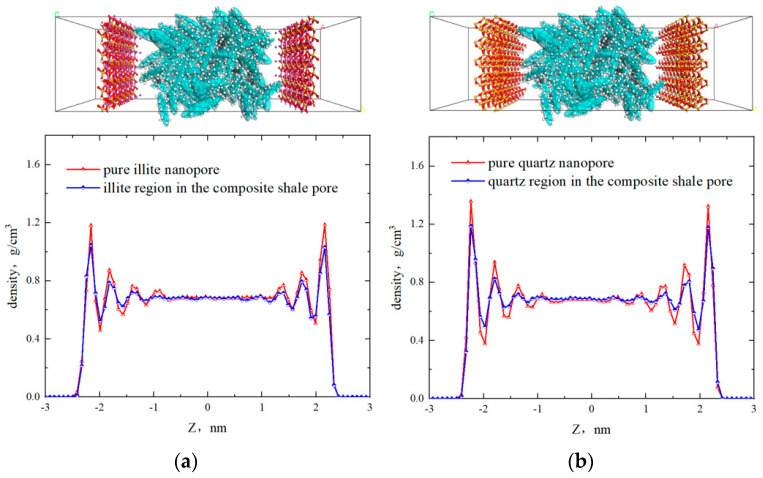
Density distribution of fluid in 5 nm pure illite (**a**) and pure quartz (**b**) nanopore.

The one-dimensional density curve of octane was axisymmetric. The first, second, third, and fourth adsorption layers were located from the surface to the center of the pore, with peak densities of 1.10 g/cm^3^, 0.79 g/cm^3^, 0.71 g/cm^3^, and 0.69 g/cm^3^, respectively. The thickness of each adsorption layer was approximately 4.5 Å, which was close to the width of the octane molecules. The two-dimensional density showed the octane distribution in different regions of the shale surface. Although there were four symmetrical adsorption layers in the quartz and illite regions, the adsorption layer density of octane in the quartz region was higher than that in the illite region, which was essentially due to the varying adsorption capacities of quartz and illite for octane molecules. To clarify the distribution characteristics of octane in homogeneous and heterogeneous shale nanopores, 5 nm pure quartz and illite slits were constructed, respectively. The model dimensions and fluid molecules numbers were consistent with the 5 nm heterogeneous model. The unit length in the z-direction (Δ*Z*) was also 0.08 nm. The density distributions in the pure quartz and illite nanopores were calculated as shown in Figure 4. In the pure illite pores, the density curve exhibited a symmetrical distribution with four adsorption layers, and the peak densities were 1.185 g/cm^3^, 0.875 g/cm^3^, 0.765 g/cm^3^, and 0.735 g/cm^3^. In the heterogeneous shale nanopore, the peak densities of the four adsorption layers in region I were 1.055 g/cm^3^, 0.795 g/cm^3^, 0.725 g/cm^3^, and 0.695 g/cm^3^. In pure quartz pores, the peak densities of the four adsorption layers of octane were 1.355 g/cm^3^, 0.945 g/cm^3^, 0.775 g/cm^3^, and 0.725 g/cm^3^. In the heterogeneous shale nanopore, the adsorption layer peak densities of region Q were 1.195 g/cm^3^, 0.835 g/cm^3^, 0.725 g/cm^3^, and 0.705 g/cm^3^. This implied that the peak densities of each adsorption layer in both region I and region Q were lower than that in the homogeneous nanopores, which could be attributed to the competitive adsorption of quartz and illite in heterogeneous pores. The interaction between the two mineral surfaces resulted in lower adsorption phase densities in the heterogeneous nanopore compared with the homogeneous nanopore. Therefore, the current calculations of hydrocarbon adsorption based on homogeneous pores might overestimate the adsorption capacity.

From Figure 3, the velocity distribution of octane shows a parabolic shape; this was because the adsorption effect was stronger in the near-wall region, resulting in a lower flow rate than at the center of the pore. The two-dimensional velocity distribution revealed that the octane velocities in the two minerals’ surfaces were different, with flow rates of 0.51 × 10^−3^ Å/fs and 0 × 10^−3^ Å/fs at the boundary of the illite region and quartz region, respectively. Additionally, the high-velocity area in the illite region was larger than that in the quartz region. The flow rate at the center of the two regions was similar because the bulk fluid was only affected by internal friction.

**Figure 5:**
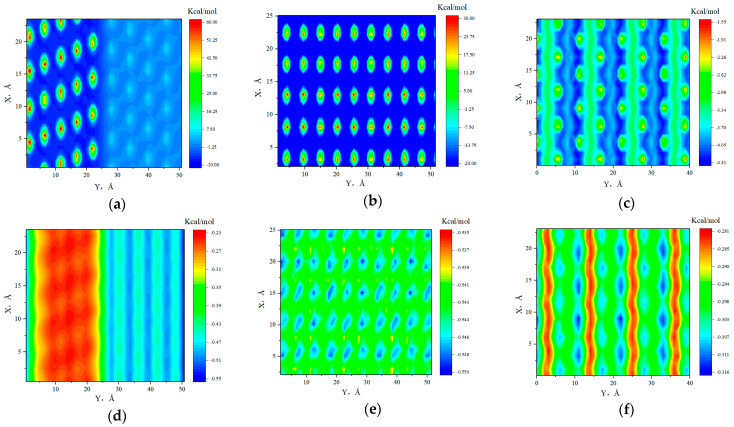
IED between C atom and heterogeneous shale surfaces (5 nm). (**a**) IED of the first adsorption layer. (**b**) IED of the first adsorption layer on the pure quartz surface. (**c**) IED of the first adsorption layer on the pure illite surface. (**d**) IED of the second adsorption layer. (**e**) IED of the second adsorption layer on the pure quartz surface. (**f**) IED of the second adsorption layer on the pure illite surface.

Figure 5 shows the IED between the C atom (octane) and the surfaces of heterogeneous shale. Figure 5a,d represent the two-dimensional potential energy distributions when the C atom was located at heights of 2.25 Å and 6.75 Å from the surface, respectively. These heights corresponded to the positions of the density peaks for the first and second adsorption layers in the 5 nm slit. In Figure 5, negative and positive interaction energy represent attraction and repulsion, respectively [68]. There was a significant gap in the IED between region Q (right) and region I (left). From Figure 5a, there is a strong repulsive interaction between the K^+^ (illite surface) and the alkane molecules in the first adsorption layer, with interaction energies reaching above 60.0 kcal/mol. Apart from the K^+^, all other positions in region I showed attractive interactions with the octane molecules, with interaction energies being less than −10.0 kcal/mol. The interaction energy between octane and the hydroxyl (quartz surface) was approximately 6.5 kcal/mol, while the interaction energy with the rest of the surface was about −3.0 kcal/mol. This indicated that hydroxyl exhibited a repulsive interaction with octane molecules, while the rest of the surface showed attractive interactions, and both the repulsive and attractive forces were weaker than those on the illite surface. Overall, the alkane adsorption was influenced by the collective effect of repulsive and attractive forces from the surface. Compared with the quartz surface, the K^+^ on the illite surface was distributed intensively, and the repulsion of K^+^ on octane molecules was very obvious, resulting in the stronger adsorption capacity of alkane in region Q than in region I. From Figure 5d, when octane molecules were located at the second adsorption layer, the repulsive effects of K^+^ and hydroxyl groups were weakened due to the greater distance to the surfaces. Additionally, the attractive forces from other positions on the surface also decreased as the distance increased, but the reduction in attractive forces was much smaller than that in repulsive forces. Both regions of the heterogeneous shale surface still exhibited attractive interactions with alkane molecules, especially region Q. Therefore, the adsorption capacity for alkanes of region Q was stronger than that of region I.

Figure 5b,c,e,f represent the interactions between the C atom (octane) and the shale surfaces at the first and second adsorption layers on pure quartz and illite surfaces. In the first adsorption layer, the hydroxyl on the pure quartz surface exhibited repulsive forces with alkane molecules, while all other positions showed strong attractive forces with interaction energies of approximately −23.5 kcal/mol. In contrast, the pure illite surface showed attractive forces, with interaction energies ranging from −1.55 kcal/mol to −4.5 kcal/mol, which were obviously weaker than those of quartz surface. Therefore, the density of the adsorption phase in region Q was higher than that in region I. At the second adsorption layer, the alkane molecules were farther away from the surfaces, resulting in a significant decrease in the interaction energies between the quartz surface and alkane, showing weaker attractive forces. The influence of K^+^ on the surface–octane interaction reduced, and the illite–octane interaction was slightly lower than the quartz–octane interaction. As a result, the adsorption phase density of the second adsorption layer in region Q remained relatively high. However, the difference in adsorption phase density between the two regions was smaller than that observed at the first adsorption layer.

### 2.2. Effect of Pore Size on the Hydrocarbon Transportation in Heterogeneous Shale Pores

**Figure 6:**
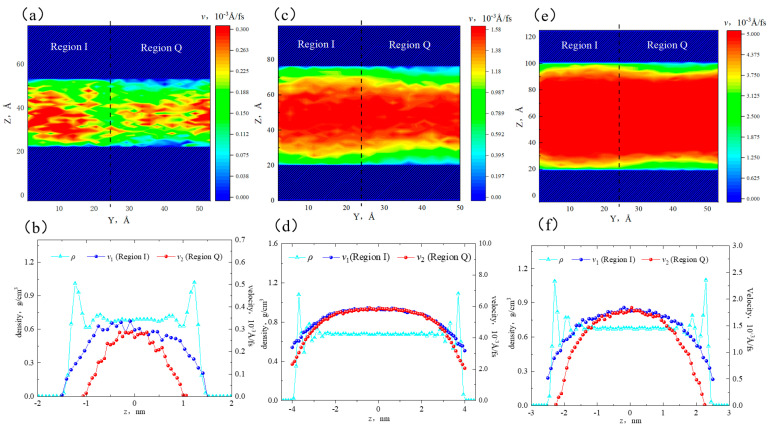
Velocity distribution of octane in different apertures: (**a**) two-dimensional velocity distribution in the 3 nm aperture, (**b**) velocity distribution of different regions in the 3 nm aperture, (**c**) two-dimensional velocity distribution in the 5 nm aperture, (**d**) velocity distribution of different regions in the 5 nm aperture, (**e**) two-dimensional velocity distribution in the 8 nm aperture, and (**f**) velocity distribution of different regions in the 8 nm aperture.

Figure 6a,c,e show that when the pore width was 3 nm, the velocity distribution in different regions showed heterogeneity. The high-velocity area in region Q was smaller, while the high-velocity area in region I was larger. In the near-wall region, the flow rate in region I was higher than that in region Q. This was because the fluid in region Q was adsorbed on the surface, leading to a decrease in flow capacity. In the 5 nm pore, the flow rate distribution became more uniform, with an increase in the high-velocity area. However, there were still velocity differences between the two sides in the near-wall region. In the 8 nm pore, the flow rate distribution showed a distinct interface, with a further increase in the high-velocity area. The velocity in the bulk region was uniformly continuous. The low-velocity area in region Q was still larger than that in region I. It is worth noting that in the 5 nm and 8 nm pores, there coexisted adsorbed and bulk fluid, while in the 3 nm pore, only adsorbed fluid was present, and there was no bulk fluid. 

**Figure 7:**
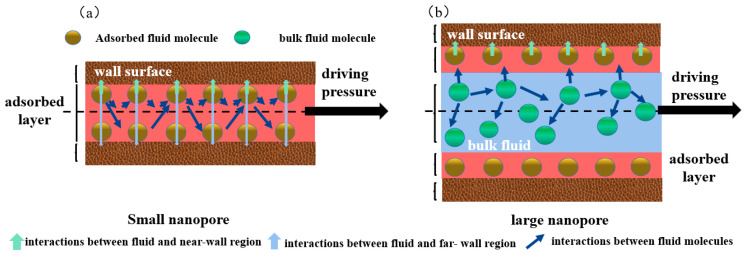
Schematic diagram of fluid distribution and force analysis in different apertures. (**a**) the forces acting on fluid molecules in small pores, (**b**) the forces acting on fluid molecules in large pores.

Figure 7 shows the fluids distribution and force analysis within different apertures. The forces acting on fluid molecules can be classified into three categories: the interaction between fluid and near-wall (INW), the interaction between fluid and far-wall (IFW), and the interaction between fluid molecules (IFF). In smaller pores, as depicted in Figure 7a, fluid molecules were subject to opposing force from INW and IFW, which partially cancelled out the INW force. Moreover, the solid–liquid interaction was stronger than the liquid–liquid interaction, resulting in a denser arrangement of fluid molecules. All fluid molecules adhered at the pore surfaces, and there was no bulk fluid. As the aperture increased, the alkane molecules in the center of the pore became farther away from the surface, and the force exerted by the surface on the fluid gradually weakened or even disappeared, as shown in Figure 7b. Free oil appeared in the center of the pore. The bulk fluid was only affected by the intermolecular force, with a density of 0.68 g/cm³, which was in good agreement with National Institute of Standards and Technology (NIST) predictions. Additionally, the bulk fluid prevented the adsorbate fluid from being influenced by the far-wall and enhancing the near-wall forces. Therefore, when the aperture exceeded 5 nm, the peak density and the number of adsorption layers were higher than those at 3 nm; 3 nm corresponded to the lower limit of movable oil within the pore.

Figure 6b,d,f depict that in the 3 nm pore, the flow rate of octane molecules in region I was higher than that in region Q. The peak velocities were 0.32 × 10^−3^ Å/fs and 0.26 × 10^−3^ Å/fs. At the boundaries, the velocity of region I was greater than 0, while there was a sticky layer in region Q. This was because all alkane molecules in both regions of the 3 nm slit were affected by the surface. Additionally, quartz had a higher adsorption capacity for alkane, resulting in a stronger restriction on the octane flow in region Q. In the 5 nm and 8 nm pores, the bulk fluid was not influenced by the surfaces and was only subjected to internal friction forces. Therefore, the flow rate of bulk fluid in both regions remained consistent. In the 5 nm pore, the flow rates at the boundaries of region I and region Q were 0.51 × 10^−3^ Å/fs and 0, respectively. In the 8 nm pore, the flow rates were 3.20 × 10^−3^ Å/fs and 2.03 × 10^−3^ Å/fs. That is to say, as the pore size increased, the velocities at the boundaries increased. When the pore size expanded to 8 nm, the sticky layer in region Q disappeared, and all fluid participated in the flow. Therefore, the flow rate was stronger in large pores than that in smaller pores.

**Figure 8:**
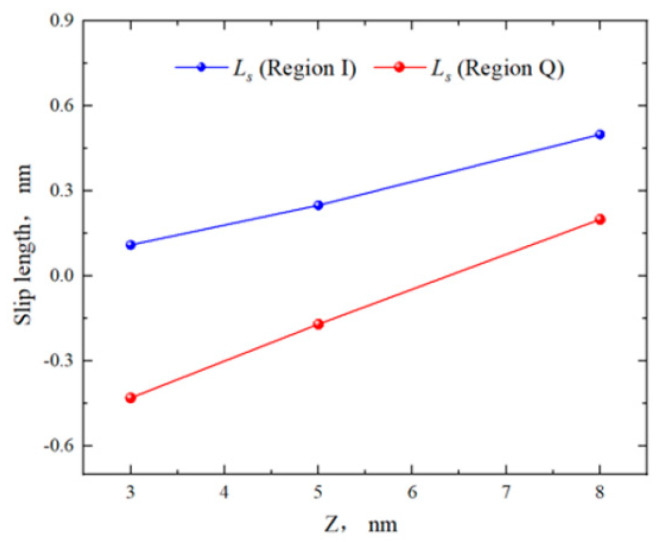
Slip lengths of octane in region Q and region I for different pore sizes.

**Figure 9:**
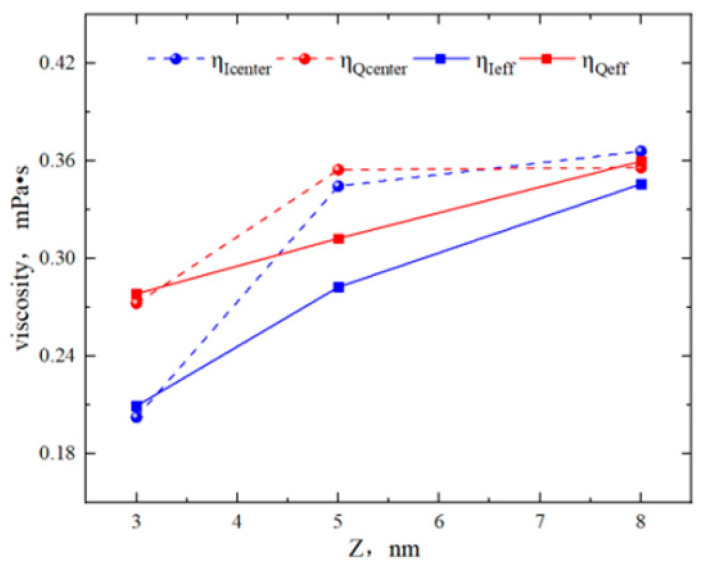
Viscosity of octane in region Q and region I for different pore sizes.

From Figure 8, the fluid slip lengths of region Q in the 3 nm, 5 nm, and 8 nm pores were −0.43 nm, −0.17 nm, and 0.2 nm, respectively. The slip lengths in region I were 0.11 nm, 0.25 nm, and 0.5 nm. As the pore size increased, the boundary of region I always exhibited “positive slip”, while the boundary of region Q gradually transitioned from “negative slip” to “positive slip”. The slip lengths in both regions increased, with the slip length in region Q remaining smaller than that in region I. From Figure 9, in the 3 nm pore, there was a significant difference in the viscosity of bulk fluid (*η*_center_) between the two regions. In the 5 nm and 8 nm pores, the average viscosity (*η*_eff_) was similar. This was because in the 5 nm and 8 nm pores, the *η*_center_ was the fitting result of the bulk fluid velocity, which was almost unaffected by the surfaces. Comparing the *η*_eff_ of the two regions for different pore sizes, it was found that the *η*_eff_ of region Q was greater than that of region I. This may be due to the stronger adsorption capability of quartz more significantly restricting the octane flow, weakening the shear of the fluid in that region and thus increasing the viscosity.

### 2.3. Effect of Temperature on the Hydrocarbon Transportation in Heterogeneous Shale Pores

**Figure 10:**
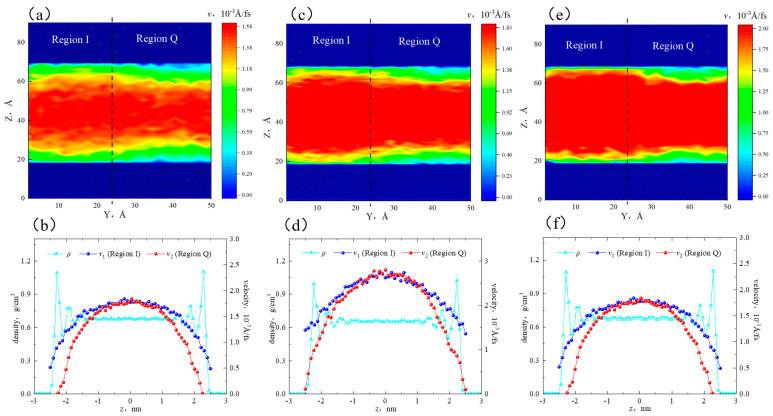
Velocity distribution of octane in different temperatures: (**a**) two-dimensional velocity distribution at 360 K, (**b**) velocity distribution in different regions at 360 K, (**c**) two-dimensional velocity distribution at 380 K, (**d**) velocity distribution in different regions at 380 K, (**e**) two-dimensional velocity distribution at 400 K, and (**f**) velocity distribution in different regions at 400 K.

Figure 10a,c,e show that with increasing temperature, the thermal motion of fluid molecules intensified, the flow rate in both regions increased, and the velocity distribution at the center of the pore became more uniform. The high-velocity areas expanded, and the low-velocity areas in the near-wall region reduced. Compared with 360 K, the low-velocity area in region I significantly reduced at 380 K, and the sticky layer disappeared in region Q. At 400 K, the velocity further increased, and there were only a few low-velocity areas near the boundary in region I. The flow rate at the boundary of region Q remained relatively unchanged. It was possible that factors such as pore size and pressure gradient, in addition to temperature, were influencing the fluid flow within this region. Figure 10b,d, and f illustrates that the flow rates at the boundary of region I at 360 K, 380 K, and 400 K were 0.50 × 10^−3^ Å/fs, 0.73 × 10^−3^ Å/fs, and 1.35 × 10^−3^ Å/fs, respectively. The flow rates in region Q were 0 Å/fs, 0 Å/fs, and 0.27 × 10^−3^ Å/fs. This indicated that the slip velocity increased as the temperature rose. The boundary of region I always exhibited “positive slip”, while the boundary of region Q transitioned from “negative slip” to “positive slip”. The velocities at the boundary of region Q were consistently lower than those in region I, which was due to the stronger adsorption of alkane molecules by quartz. The temperature could not shield the difference in adsorption capacity.

**Figure 11:**
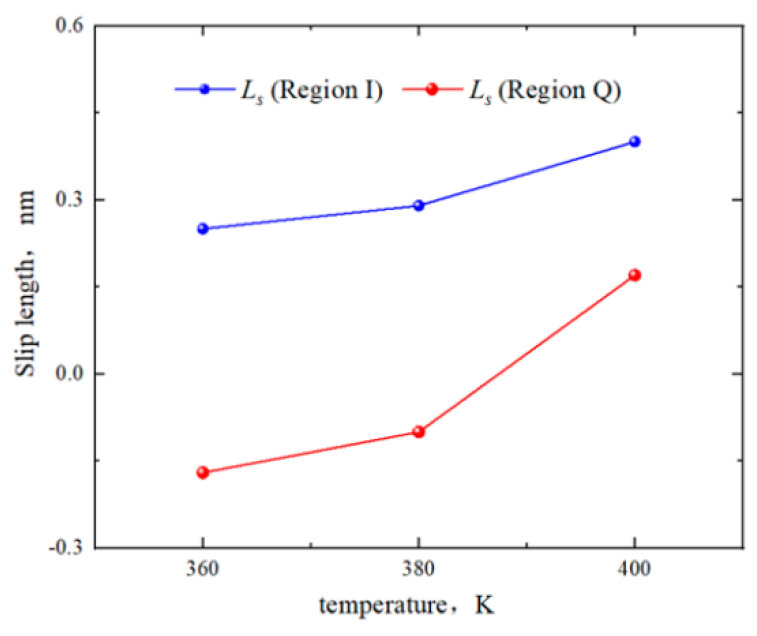
Slip lengths of octane in region Q and region I for different temperatures.

**Figure 12:**
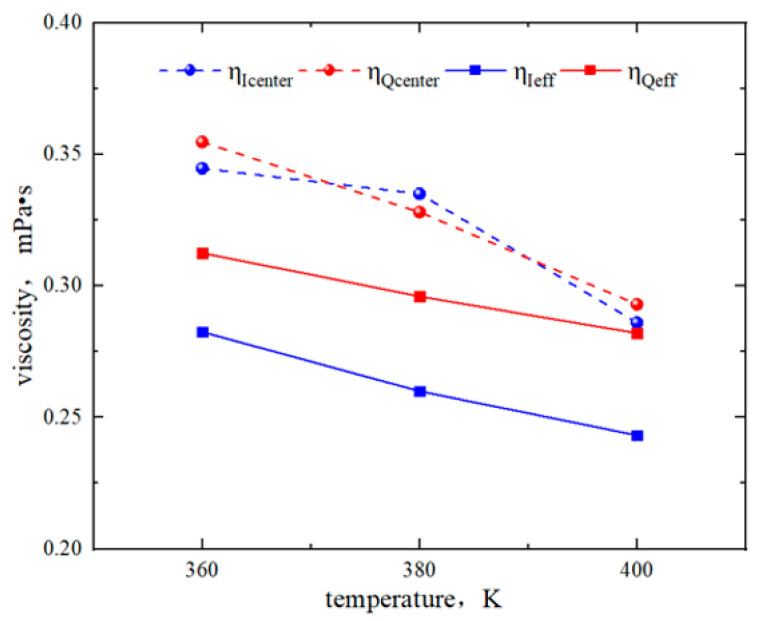
Viscosity of octane in region Q and region I for different temperatures.

In Figure 11, the slip lengths of region Q and region I are −0.17 nm and 0.25 nm at 360 K, −0.1 nm and 0.29 nm at 380 K, and 0.17 nm and 0.4 nm at 400 K. The slip lengths increased with the temperature rises. The boundary of region I maintained “positive slip”. The boundary of region Q exhibited “negative slip” below 380 K and turned to “positive slip” above 380 K. In Figure 12, the *η*_center_ of region Q at 360 K, 380 K, and 400 K is 0.35 mPa·s, 0.33 mPa·s, and 0.29 mPa·s, respectively, with the *η*_eff_ of 0.319 mPa·s, 0.30 mPa·s, and 0.28 mPa·s, respectively. In region I, the *η*_center_ is 0.34 mPa·s, 0.35 mPa·s, and 0.29 mPa·s, respectively. The *η*_eff_ is 0.28 mPa·s, 0.26 mPa·s, and 0.24 mPa·s. Thus, the viscosity of octane in both regions decreased with increasing temperature, and the *η*_center_ in region Q and region I remained the same due to the bulk fluid not being influenced by the surfaces.

### 2.4. Effect of Pressure Gradient on the Hydrocarbon Transportation in Heterogeneous Shale Pores

**Figure 13:**
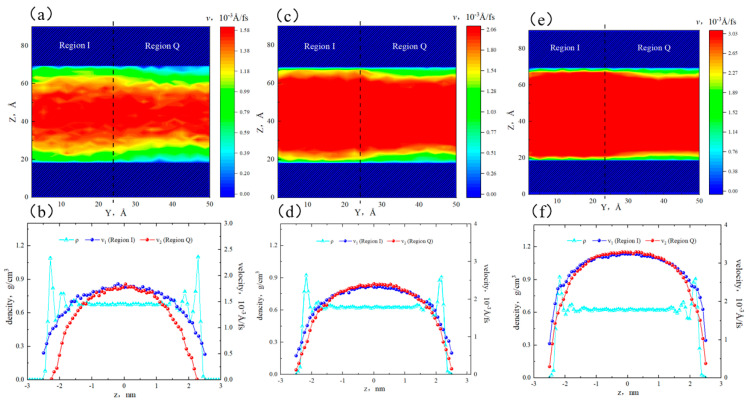
Velocity distribution of octane in different pressure gradients for 5 nm pore: (**a**) two-dimensional velocity distribution at 0.002 Kcal/(mol∙Å), (**b**) velocity distribution in different regions at 0.002 Kcal/(mol∙Å), (**c**) two-dimensional velocity distribution at 0.0025 Kcal/(mol∙Å), (**d**) velocity distribution in different regions at 0.0025 Kcal/(mol∙Å), (**e**) two-dimensional velocity distribution at 0.003 Kcal/(mol∙Å), and (**f**) velocity distribution in different regions at 0.003 Kcal/(mol∙Å).

Figure 13a,c,e show that as the pressure gradient increased, the high-velocity areas in the center of the pore expanded, while the low-velocity area in the near-wall region reduced. When the pressure gradient was 0.002 Kcal/(mol∙Å), the low-velocity area in region Q was significantly greater than that in region I. When the pressure gradient was 0.003 Kcal/(mol∙Å), the heterogeneity of the velocity distribution decreased. Figure 13b,d,f showed that at 0.002 Kcal/(mol∙Å), there was a significant difference in the velocity distribution between the two regions. Region Q exhibited a sticky layer, and the flow rate of octane at the boundary of region I was 0.51 × 10^−3^ Å/fs. At 0.0025 Kcal/(mol∙Å), the octane velocities at the boundary of region Q and region I were 0.12 × 10^−3^ Å/fs and 0.52 × 10^−3^ Å/fs, respectively. This implied that there was a significant velocity variation at the boundary of the quartz region. When the pressure gradient reached 0.003 Kcal/(mol∙Å), the octane velocities at the boundary of region Q and region I were 0.3 × 10^−3^ Å/fs and 0.89 × 10^−3^ Å/fs, respectively. The boundary flow rate significantly increased at both regions. Additionally, the velocity distribution curves of the two regions almost overlapped at 0.003 Kcal/(mol∙Å), which could have been due to the dominant factor being the pressure gradient at this stage; the effects of other factors such as temperature and pore size became much smaller compared with the pressure gradient.

**Figure 14:**
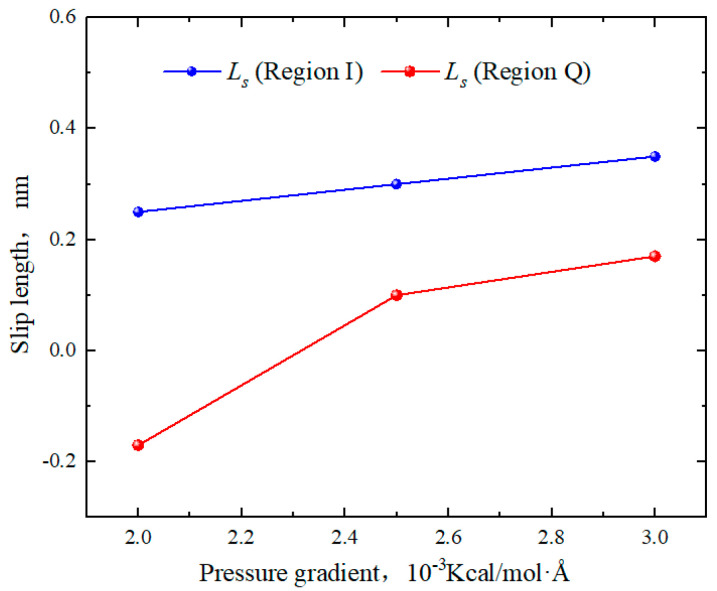
Slip lengths of octane in region Q and region I for different pressure gradients.

**Figure 15:**
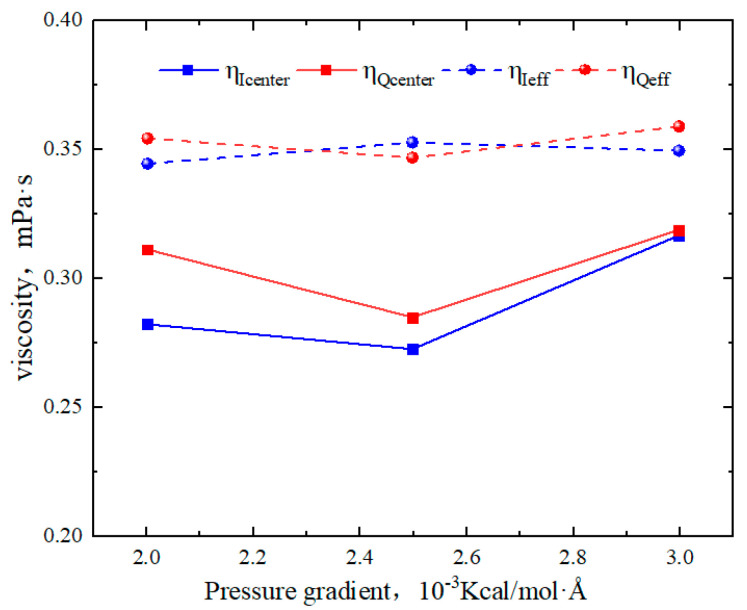
Viscosity of octane in region Q and region I for different pressure gradients.

As shown in Figure 14, the slip lengths of octane in region Q under various pressure gradients were −0.17 nm, 0.1 nm, and 0.17 nm, respectively. In region I, the slip lengths were 0.25 nm, 0.3 nm, and 0.35 nm. Therefore, as the pressure gradient increased, the slip lengths in region I exhibited a linear increase. The slip lengths in region Q initially increased sharply, transforming from “negative slip” to “positive slip”. From Figure 15, the viscosity of bulk fluid in both regions remained around 0.35 mPa·s and showed little variation with the pressure gradient. At lower pressure gradients, the viscosity of octane in region Q was higher than that in region I. As the pressure gradient increased, the average viscosity in both regions tended to become similar. This phenomenon occurred because when the pressure gradient reached 0.003 Kcal/(mol∙Å), the low-velocity areas at the boundaries reduced, and the influence of the surface on the hydrocarbon flow became much smaller compared with the pressure gradient. As a result, the velocity distributions of octane in both regions became almost identical.

### 2.5. Hydrocarbon Transportation in Heterogeneous Shale Pores under Aqueous Conditions

#### 2.5.1. Micro-Distribution of Oil–Water Two-Phase Region in Heterogeneous Shale Pores

**Figure 16:**
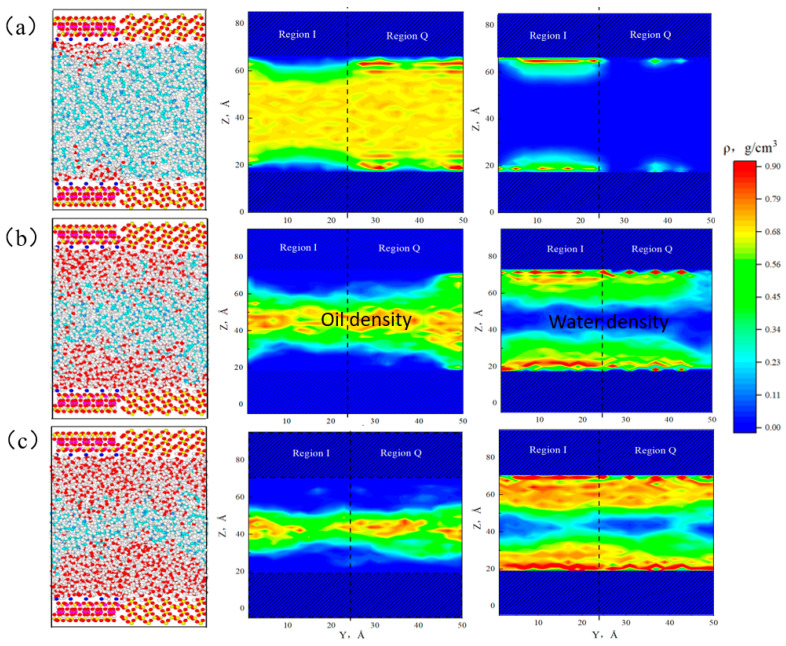
The equilibrium configuration and two-dimensional densities of oil–water two-phase region in heterogeneous shale pore at water content of 10% (**a**), 50% (**b**), and 70% (**c**).

Shale reservoirs contain original irreducible water, and fracturing fluid is also injected into the formation during the hydraulic fracturing, leading to shale reservoirs being commonly situated in aqueous conditions. Water molecules will seriously affect the fluid flow characteristics. Oil–water two-phase models are established within the shale pores at different water contents. The shale surface model refers to Section 2.1, and the equilibrium configuration and two-dimensional densities of the oil–water two-phase region in the heterogeneous shale pore are shown in Figure 16. 

When the water content was 10%, the near-wall region was a two-phase (oil–water) region, oil molecules were distributed throughout the pores, and both oil and water phases exhibited two distinct adsorption layers. However, the adsorbed water phases in these two regions did not saturate completely, and water molecules spread out on the surface of region I in the manner of “liquid film”, while there were only a small number of water molecules on the surface of region Q. This may be attributed to the stronger hydrophilicity of illite, which exhibited a distinct advantage in the competitive adsorption for water molecules. The oil phase exhibited the opposite behavior, with high-density areas mostly concentrated on the surface of region Q and low-density areas distributed on region I. Compared with the surface of illite, quartz exhibited an “oil-wet” property. When the water content was 50%, water molecule accumulation was more pronounced in region I, with its surface being entirely enveloped by water molecules, and the remaining water molecules were insufficient to saturate the quartz surfaces; octane molecules were also adsorbed on the quartz surfaces, leading to an oil–water two-phase region in the near-wall region of quartz surfaces. The numbers of adsorption water layers increased compared with that at 10% water content, resulting in an enlargement of the oil–water two-phase region. At 70% water content, the near-wall region of regions I and Q was completely occupied by water molecules. Unlike the case of 50% water content, at 70% water content, there was a small amount of water at the center of the pore. This was because the adsorbed water phase reached saturation, there were still remaining water molecules that coexisted with the oil phase in a free state in the center of the pore, and the fluids were distributed in a layered structure of “water–two-phase region (oil and water)–water”. The density of the adsorbed water phase in region I remained higher than that in region Q, further confirming that illite exhibited stronger hydrophilic ability.

#### 2.5.2. Oil–Water Transportation in Heterogeneous Shale Pores

**Figure 17:**
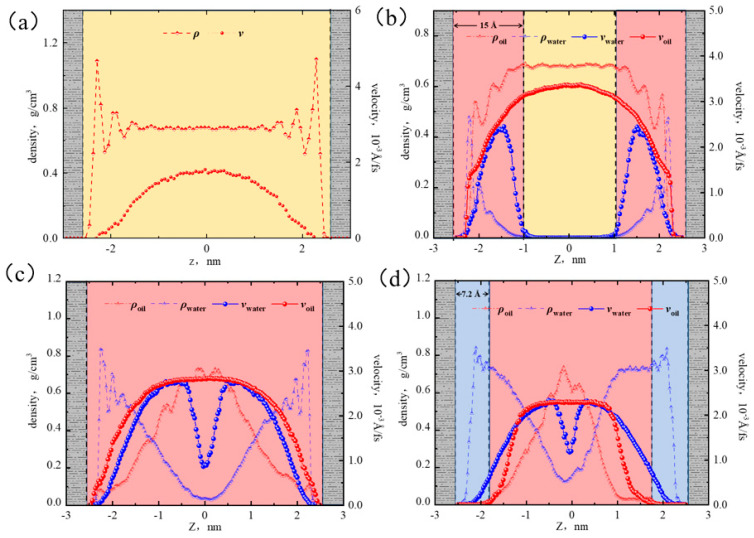
Velocity distribution of oil–water at water contents of 0% (**a**), 10% (**b**), 50% (**c**), and 70% (**d**).

Based on the non-equilibrium MDS method, the velocity distribution curves of oil and water were calculated under the water contents of 0%, 10%, 50%, and 70%. As shown in Figure 17, the dashed line represents the density, the solid line represents the velocity, the pink represents the oil-water two phase, the blue represents the water phase, the yellow represents the oil phase, and the gray represents the shale surface. Figure 17 illustrates that at a low water content, the velocity distribution of the oil phase was parabolic, and the peak velocity was 3.372 × 10^−3^ Å/fs, which was significantly higher than the velocity at 0% water content (1.782 × 10^−3^ Å/fs). This was because the shale surface was hydrophilic, where water molecules aggregated at the pore surface, weakening the alkane–surface interaction. As a result, the flow resistance of octane molecules was reduced, and the flow rate was increased. The velocity of the water phase was 0 Å/fs at the boundary and reached its peak velocity at a distance of 10 Å from the surface, then gradually decreased, and at a distance of 15 Å from the surface, the velocity decreased to 0 Å/fs. At 50% water content, the water phase velocity at the wall was 0 Å/fs, and a sticky layer with a thickness of 2.5 Å was formed in the near-wall region, which restricted the movement of water molecules. Since there were only a few water molecules present at the center of the pore, the water velocity distribution curve showed a decreasing funnel shape. The oil velocity distribution still followed a parabolic shape, with the velocity at the boundary being 0 Å/fs, but there was no sticky layer. Compared with 10% water content, the oil velocity was lower at 50% water content. This was because the oil–water two-phase region was larger, and the oil flow was significantly affected by the water. At high water content (70%), the thickness of the sticky layer increased to 4 Å, and there was a smaller amount of free water molecules appearing in the center of the pore. At a distance of 7.2 Å from the wall, the water molecules reached an adsorbed saturated state, completely shielding the octane–surface interaction, resulting in the absence of the oil phase. Due to the large water phase region, the oil velocity in the oil–water two-phase region was lower than the water velocity.

**Figure 18:**
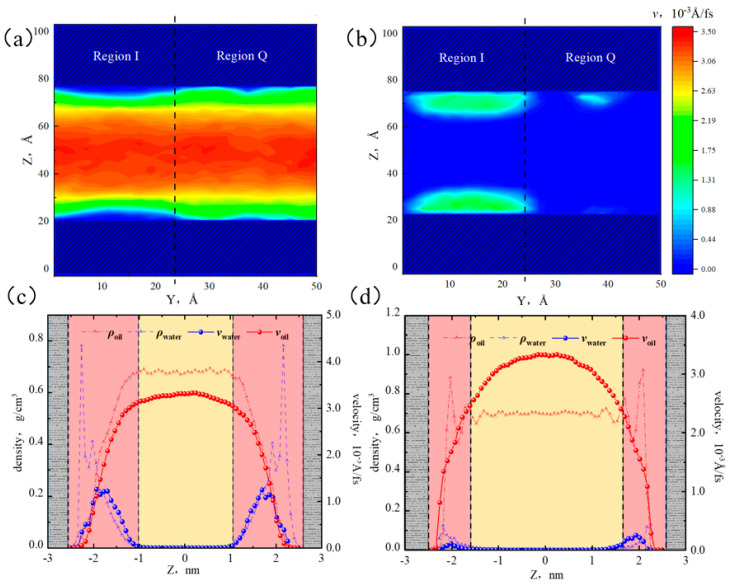
Velocity distribution of oil and water at 10% water content: (**a**) two-dimensional velocity distribution of octane, (**b**) two-dimensional velocity distribution of water, (**c**) oil and water velocity distribution in region I, and (**d**) oil and water velocity distribution in region Q.

From Figure 18a, when the water content was 10%, the high-velocity area of the oil phase was mainly distributed in the center of the pore. Compared with 0% water content, the oil flow rate difference in region I and Q was significantly smaller, and the velocity distribution of oil was more uniform. This was because the water molecules adsorbed on the surfaces exerted a certain balancing effect on the alkane–surface interactions. The low-velocity area of the oil phase was distributed in the near-wall region, showing clear heterogeneity. According to Figure 18b, the water phase was mainly concentrated on the surfaces of region I, with only a few water molecules in region Q. The strong adsorption of water molecules by the surface resulted in a lower water flow rate.

Figure 18c,d show that in the near-wall region, the velocity distributions of water and oil molecules in region I were similar, while the velocity of water was lower than that of oil in region Q. At the boundary, the velocities of octane molecules in regions I and Q were 0 and 1.10 × 10^−3^ Å/fs, respectively. This indicated that at lower water content, the presence of water phase in heterogeneous inorganic nanopores would limit the oil flow in region I but promoted the oil flow in region Q. This was because the fluid in the near-wall region of region I was mixed distributed, leading to a decrease in the oil flow rate influenced by water molecules. Although the quartz surface was also water-wet, the hydrophilic ability of quartz was lower than that of illite, and only a small number of water molecules gathered on the surface of region Q. However, the water molecule–quartz interaction weakened the adsorption for octane molecules. Therefore, compared with the case of 0% water content, the flow rate of octane at the boundary in region Q was significantly higher.

**Figure 19:**
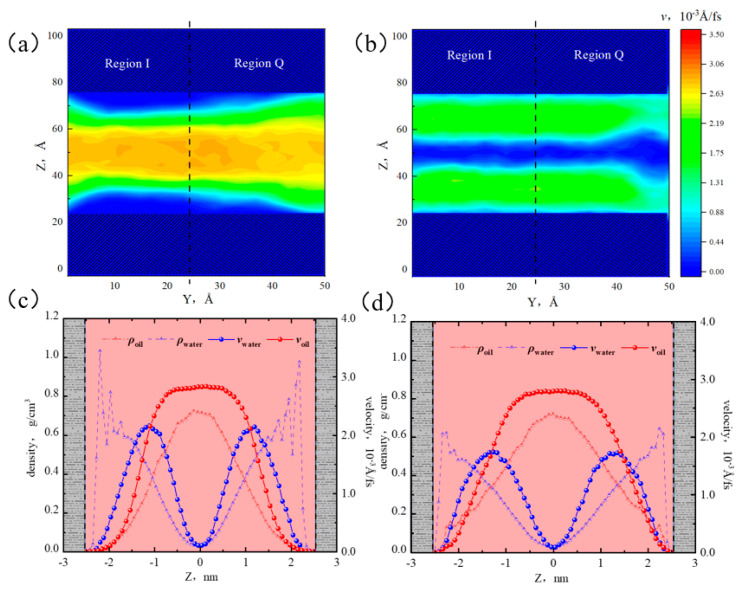
Velocity distribution of oil and water at 50% water content: (**a**) two-dimensional velocity distribution of octane, (**b**) two-dimensional velocity distribution of water, (**c**) oil and water velocity distribution in region I, and (**d**) oil and water velocity distribution in region Q.

From Figure 19a,b, when the water content was 50%, the oil flow rate at the center of the pore was significantly reduced, and the heterogeneity of the flow rate at the boundaries in both regions became more pronounced. In region I, the low-velocity area of the oil phase in the near-wall region increased, and the velocity distribution of the water phase showed a regular layered structure. In region Q, the low-velocity area of oil phase on the left was larger than that on the right, and the distribution of water phase on the left was relatively regular compared with that on the right. Based on the results of Section 2.5.1, it can be inferred that at 50% water content, the adsorption of the water phase on the surface of region Q had not reached saturation, leading to an uneven distribution of oil and water on the surface of region Q.

Figure 19c,d show that the peak velocities of water and oil in region I were 2.16 × 10^−3^ Å/fs and 2.82 × 10^−3^ Å/fs, respectively. Compared with 10% water content, the water flow rate in region I increased significantly, while the oil flow rate decreased. Because of the water content increases, the oil–water two-phase region expanded, and the flow rates of oil and water mutually influenced each other and tended to become similar. Due to the strong adsorption of illite on water molecules, a non-flowing sticky water layer began to appear at the boundary of region I. However, there was no sticky water layer at the boundary in region Q; because the water phase had not reached saturation in this region, and the water distribution was uneven in the near-wall region, the range of the oil phase was larger, resulting in the water flow rate being lower than that in region I.

**Figure 20:**
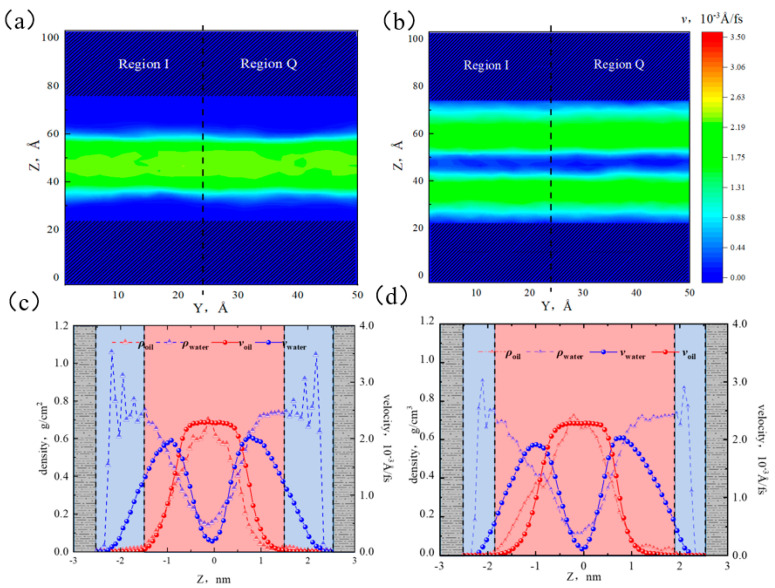
Velocity distribution of oil and water at 70% water content: (**a**) two-dimensional velocity distribution of octane, (**b**) two-dimensional velocity distribution of water, (**c**) oil and water velocity distribution in region I, and (**d**) oil and water velocity distribution in region Q.

From Figure 20a,b, the velocity distributions in regions I and Q were almost identical, and the layered structure of oil–water velocities was more distinct. The oil phase was only present at the center of the pore, with a lower velocity compared with 50% water content. From Figure 20c,d, the peak velocities of the water and oil were 2.16 × 10^−3^ Å/fs and 2.28 × 10^−3^ Å/fs, respectively. Compared with the case of 50% water content, the water velocity increased, while the oil velocity decreased. This was because as the water content increased, the adsorbed water layer became thicker, and the interaction between water molecules and far-wall was weaker, resulting in a higher flow rate. Additionally, the adsorbed water layers in both regions were sufficiently thick because the adsorbed water phases had reached a saturated state. The interaction between the pore surface and the octane molecules weakened. The oil flow was only influenced by the driving force and the intermolecular forces. The friction between the water layer and the oil layer caused the flow rates of the oil and water to be similar.

Based on the velocity distribution of oil and water under different water contents, the volumetric fluxes in each region and volumetric flux percentage of oil are calculated by Equations (1) and (2):

(1) Q=∫vds

where *Q* is the flux, v is the flow rate, and *s* is the cross-sectional area of the pore.

(2) $$N=\frac{Q_{o}}{\left(Q_{o}+Q_{w}\right)}$$

where *N* is the volumetric flux percentage of oil, and *Q*_o_ and *Q*_w_ are the volumetric fluxes of oil and water, respectively.

**Figure 21:**
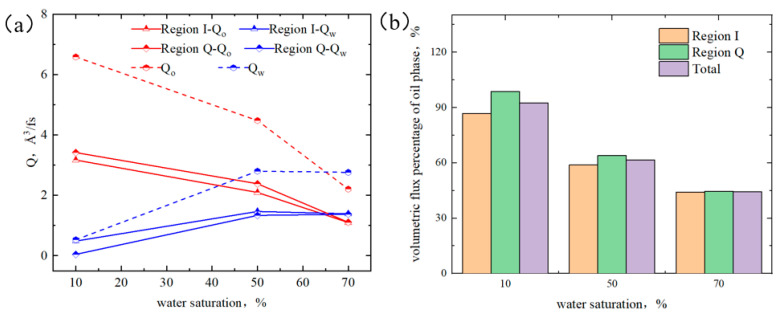
Oil and water fluxes (**a**) and volumetric flux percentage of oil (**b**) under different water saturation.

As shown in Figure 21a, when the water content was low, the water flux in region I was higher than that in region Q, and the oil flux was lower than that in region Q. As the water content increased, the difference in oil and water fluxes between the two regions became smaller. When the water content was 70%, both quartz and illite regions were all saturated with water; therefore, the oil–water flow fluxes in both regions were consistent. From Figure 21b, at 10% water content, the oil flux percentages in regions I and Q were 87% and 99%, respectively. At 50% water content, the flux percentages in regions I and Q were 59% and 64%, respectively, showing a downward trend, and the difference between the two regions was narrowing. This was because the adsorbed water layer weakened the alkane–surface interaction but was not sufficient to completely shield the interaction until the water content reached 70%; the oil flux percentages in both regions were all 44%. 

## 3. Models and Methods

### 3.1. Modeling

Inorganic minerals are important components of shale matrix, including quartz, calcite, and clay minerals [27]. Among the clay minerals, illite, MMT, and interstratified illite/MMT are the predominant constituents, with a small proportion of interstratified chlorite/MMT. According to the previous study of the research group [69], the whole rock analysis and clay mineral analysis were performed on rock samples of Member Qing 1 in the Songliao Basin using XRD. The results indicated that the primary components of inorganic matter were quartz and clay minerals. The average contents of clay and quartz minerals were 37.7% and 36.12%, respectively. Illite accounted for the most significant proportion of clay minerals, with a 74.1% average content. Therefore, it was considered that quartz and illite were the main components of inorganic materials in shale. Quartz primarily exists in the form of α-SiO_2_ in nature, which is an important diagenetic mineral in tight, sedimentary, and metamorphic rocks. The α-SiO_2_ is a three-dimensional structure composed of SiO_2_ tetrahedrons connected by common vertices. Silicon atoms are located in the center of the tetrahedron; 1 silicon atom bonds with 4 oxygen atoms, while 1 oxygen atom bonds with 2 silicon atoms, and oxygen atoms are on the common vertices of the silicon oxygen tetrahedron [70]. Illite is a potassium-rich silicate mica clay mineral, which is a 2:1 clay mineral composed of an Al-O octahedral layer between two Si-O tetrahedra centered on a silicon atom. We constructed the illite model based on the formula of K_x_[Si_(8-x)_Al_x_](Al_4_)O_20_(OH)_4_(x = 1) [71], and K^+^ is randomly distributed among the illite layers. The atomic coordinates and cell parameters of α-SiO_2_ and illite were from the American Mineralogist Crystal Structure Database (http://rruff.geo.arizona.edu/AMS/amcsd.php). The (0 0 1) plane was the most representative crystal plane of illite, which could be used to investigate the interaction between the external environment and the crystal plane. In addition, Chilukoti et al. [70] found that liquid alkane was more inclined to adsorb on the (1 0 0) crystal plane of α-SiO_2_; it was used as the representative plane of silica in this paper. There are various types of pore shapes in shale reservoirs; slit-shaped pores are the most common in clay. This paper studied the adsorption, diffusion, and transport of hydrocarbons based on the slit-shaped pores used by Rao et al. [72], Sun et al. [73], Underwood et al. [74], and Hao et al. [75].

(1) Shale surface model

The quartz and illite were cut, then the hydroxylate the quartz, and the hydroxylated quartz and illite were extended to the dimensions 2.73 nm × 2.09 nm × 1.12 nm and 2.89 nm × 3.01 nm × 1.12 nm, respectively. Finally, we combined them to form a heterogeneous inorganic pore surface. The simulated box size was 2.87 nm × 5.30 nm × 1.12 nm. We marked the quartz and illite regions in the heterogeneous shale as “region Q” and “region I”, respectively.

(2) Fluid molecules model

**Figure 22:**
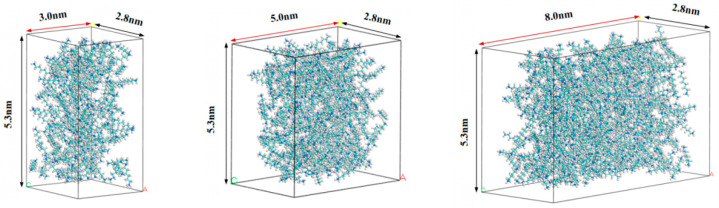
Fluid models with different pore sizes.

The chemical composition of shale oil is complex, including a large number of n-alkanes, branched alkanes, cycloalkanes, aromatic hydrocarbons and bitumen, etc. Wang et al. [76] studied the physical properties of fluids in different shales, and the results showed that the properties of the alkane mixture composed of n-CH_4_, n-C_5_H_12_, and n-C_8_H_18_ are almost the same as those of the single component n-C_8_H_18_. Therefore, n-C_8_H_18_ (octane) was selected as the shale oil model. OA, OB, and OC correspond to the lengths in the X, Y, and Z directions of the system, respectively. The OA–OB surface is the contact surface between the fluid and the shale surface. To prevent stretching or compression during the combination of the fluid and the wall, the lengths of OA and OB were consistent with the shale surface model. Therefore, the length of the fluid model OC was the pore size. We reserved a 2Å vacuum regions at both ends of the OC direction to shield periodic boundary effects and added 164, 273, and 436 octane molecules in the pores of 3 nm, 5 nm, and 8 nm, respectively; the corresponding fluid model is shown in Figure 22.

(3) Pore model

**Figure 23:**
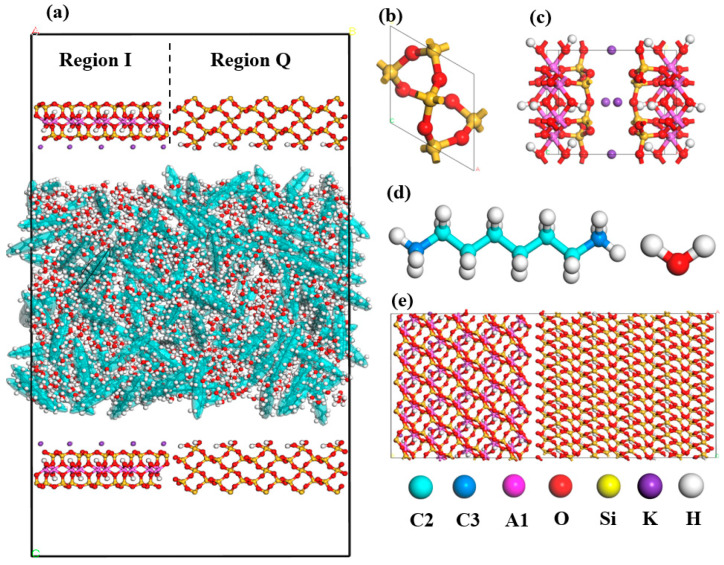
Pore and fluid model of heterogeneous inorganic shale: (**a**) nanopore model, (**b**) quartz unit cell structure, (**c**) illite unit cell structure, (**d**) alkane and water model, and (**e**) heterogeneous shale surface model.

The system energy was minimized by conjugating the gradient algorithm to avoid the atoms overlapping in pores or atomic distances being too close at the boundaries, and the positions of all atoms were adjusted to obtain a stable initial configuration. The fluid model was combined with the shale surface model for different pore sizes, and 1 nm vacuum region was added at both ends of the wall; the nanopore, the shale surface, and fluid model are shown in Figure 23.

### 3.2. Methodology 

MD simulations were conducted using LAMMPS software (Version 3 Mar 2020). The simulation details, including structure optimization, temperature, pressure control, and force field description, are shown in Table 1. 

Single-phase oil behavior simulation: The three-dimensional periodic boundary conditions were applied to the initial model. The model structure was optimized, the optimized model was relaxed for 5 ns under the NVT ensemble until the temperature reached 360 K, followed by NPT relaxation with volume fluctuations only in the Z-direction; the pressure was controlled by the Parrinello–Rahman (30 MPa). A 1.0 fs time step was set, and the last 5000 ps of data were selected as atomic trajectories, density distributions, diffusion coefficients, etc. Probe atoms were used to obtain information on different regions of heterogeneous shale, where carbon atoms and oxygen atoms represented alkane molecules and water molecules, respectively. When the probe atoms moved in the Y direction, the region from 0 Å to 24 Å represented the illite region (region I), and the region from 24 Å to 50 Å represented the quartz region (region Q).

**Figure 24:**
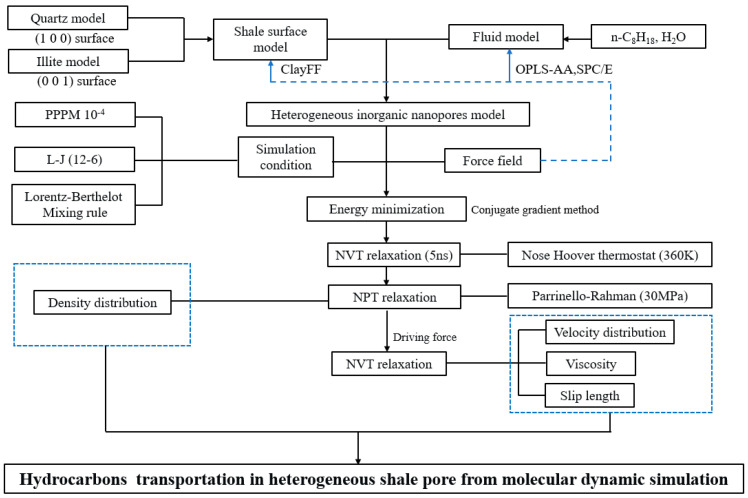
Simulation flow chart.

Oil–water two-phase behavior simulation: Oil–water two-phase systems were built with different water saturations (10%, 50%, and 70%). The SHAKE algorithm was applied to maintain the rigidity of two hydrogen–oxygen bonds and one hydrogen–oxygen–hydrogen angle [80]. The terminate tolerances of energy and force were set to 1.0 × 10^−15^ kcal/ (mol·Å) for energy minimization of the initial model, and the maximum iterations were 1000. The optimized model was relaxed for 5 ns under the NVT ensemble, followed by NPT relaxation for 5 ns. Equilibrium molecular dynamics information was collected for the system using the last 1 ns of data. We studied the two-phase transport mechanism based on the non-equilibrium molecular dynamics. An external force of 0.002 Kcal/(mol·Å) was applied to the fluid (n-C_8_H_18_, water) along the X direction, which simulated the oil–water flow in the nanopores. The model was relaxed for 10 ns under the NVT ensemble, and the last 1 ns of data were selected as the atomic trajectory and velocity distribution. Detailed parameters of the force field can be found in the references in Table 1. The van der Waals forces between different atoms were calculated using the Lorentz–Berthelot mixing rule, and the simulation flow chart is shown in Figure 24.

Interaction energy: To clarify the interaction between the hydrocarbon and the shale surface, we tested the interaction energy distribution (IED) between the shale surface and fluid molecules by the directional movement of probe atoms on the slit surface. We set the probe atom speed as 1 Å/fs; it moved uniformly above the surface along the Y direction. When the probe atom reached the boundary, it moved 1 Å along the X direction and then moved uniformly along the opposite direction of the Y; the motion was repeated until the probe atom scanned the entire surface. The atomic coordinates and energy were recorded with intervals of 1.0 fs, and the IED was calculated between the surface and the fluid molecules.

## 4. Conclusions

(1) Fluid (octane) molecules exhibited non-uniform distribution in heterogeneous inorganic nanopores, and the adsorption capacity for alkanes in quartz region was stronger than the illite region, leading to the density of the adsorbed phase in the illite region being lower than that in the quartz region. The flow rates at the boundaries of the illite and quartz region were 0.51 × 10^−3^ Å/fs and 0, respectively.
(2) In smaller heterogeneous inorganic pores (3 nm), fluid molecules were subjected to the force from both sides of the walls in opposite directions, resulting in the fluid being completely adsorbed on the wall without any bulk fluid. As the aperture increased, the bulk fluid shielded the forces from the far-wall region, the bulk fluid flowed at higher velocities, and the velocity distributions in the two regions became more uniform. The low-velocity area on the quartz region was still larger than that on the illite region.
(3) The transportation characteristics of octane in heterogeneous inorganic nanopores were significantly influenced by the temperature and pressure gradient. The quartz region was more sensitive to temperature. As the pore size, temperature, and pressure gradient increased, the boundary in the quartz region could transform “negative slip” to “positive slip”.
(4) The illite region exhibited stronger hydrophilicity than the quartz region. When the water content was low, water molecules preferentially formed a “liquid film” on the illite surface, promoting the oil flow in the quartz region. At 50% water content, the adsorption of the water phase reached saturation in the illite region, and the quartz region remained unsaturated, causing the distribution of water near the wall to be uneven. At 70% water content, the adsorbed water phases in the two regions reached a saturated state. The interaction between the wall and the octane was completely shielded, the oil flux percentages in both regions were all 44%, and a layered structure of “water–two-phase region–water” was formed.

## Figures and Tables

**Table 1:** The description of the simulation details.

Simulation Details	Description or Method (Precision)
Unit	Real
Long-range electrostatic interactions	Ewald summation method (10^−4^)
Short-range non-bonded interactions	Lennard–Jones (12–6)
Particle mesh interactions	PPPM (10^−6^)
Boundary condition	P P P
Cutoff radius	12 Å
Energy minimization	Conjugate gradient method
Temperature control	Nose Hoover thermostat
Pressure control	Parrinello–Rahman Voltage stabilizer
Relaxation ensemble	NVT + NPT
Dynamic simulation ensemble	NPT
Interactions between surface atoms	ClayFF [77]
Interactions between alkane molecules	OPLS-AA [78]
Water molecule	SPC/E [79]

## Data Availability

The data presented in this study are available in article.
